# Upregulated CANT1 is correlated with poor prognosis in hepatocellular carcinoma

**DOI:** 10.1186/s12885-023-11463-4

**Published:** 2023-10-19

**Authors:** Ting Liu, Zhi-zhao Li, Lei Sun, Kun Yang, Jia-min Chen, Xiao-yi Han, Li-ming Qi, Xin-gang Zhou, Peng Wang

**Affiliations:** 1 Department of Pathology, Beijing Ditan Hospital, Capital Medical University, No. 8 Jing Shun East Street, Chaoyang District, Beijing, 100015 China; 2 Department of Cardiovascular medicine, Beijing Ditan Hospital, Capital Medical University, No. 8 Jing Shun East Street, Chaoyang District, Beijing, 100015 China

**Keywords:** Hepatocellular carcinoma, CANT1, Prognosis, Immune cell infiltration, Immune checkpoints

## Abstract

**Background:**

CANT1, as calcium-activated protein nucleotidase 1, is a kind of phosphatase. It is overexpressed in some tumors and related to poor prognosis, but few studies explore its function and carcinogenic mechanism in hepatocellular carcinoma (HCC).

**Methods:**

The expression of CANT1 mRNA and protein was analyzed by the Cancer Genome Atlas (TCGA) database and immunohistochemistry(IHC) staining. The relationship between CANT1 expression and clinicopathology was evaluated by various public databases. The receiver operating characteristic (ROC) curve was used to assess the diagnostic accuracy of CANT1 by the area under curve (AUC). Univariate, multivariate Cox regression and Kaplan-Meier curves were applied to evaluate the predictive value of CANT1 on the prognosis of HCC. Methsurv was used to analyze gene changes and DNA methylation, and its impact on prognosis. The enrichment analysis of DEGs associated with CANT1 revealed the biological process of CANT1 based on Gene Set Enrichment Analysis (GSEA). The relationship between immune cell infiltration level and CANT1 expression in HCC was investigated using the single-sample GSEA (ssGSEA) method and the Tumor Immune Estimation Resource (TIMER) database. Finally, the association between CANT1 and immune checkpoints and drug sensitivity was also analyzed.

**Results:**

CANT1 was highly expressed in 22 cancers, including HCC, and CANT1 overexpression in HCC was confirmed by IHC. The expression of CANT1 was correlated with clinical features, such as histologic grade. Highly expressed CANT1 caused poor overall survival (OS) of HCC patients. Univariate and multivariate regression analysis suggested that CANT1 was an independent prognostic marker. Of the 31 DNA methylation at CpG sites, three CpG sites were associated with the prognosis of HCC. GSEA indicated that CANT1 was mainly involved in the cell cycle, DNA replication, and etc. Moreover, CANT1 expression was correlated with immune cell infiltration and independently associated with the prognosis of HCC patients. Finally, CANT1 expression was correlated with most immune checkpoints and drug sensitivity.

**Conclusion:**

CANT1 may be a latent oncogene of HCC, and associated with immune cells and immune checkpoints, which may assist in HCC treatment.

## Introduction

Hepatocellular carcinoma (HCC) is the third leading cause of cancer deaths worldwide and ranks sixth among diagnosed cancers [1]. In 2020, cancer deaths in China included 390,000 cases of liver cancer. Incidence and mortality rates of liver cancer were higher in China [2]. Since the early symptoms of HCC patients are unobvious, most patients are diagnosed at a later stage. Their clinical prognosis has not been significantly improved, although surgical resection and radiofrequency ablation developed greatly [3]. Therefore, it is important to explore useful biomarkers and targets for HCC prognosis to improve the diagnosis, prevention, and treatment of HCC.

Calcium-activated protein nucleotidase 1 (CANT1), as a calcium-dependent nucleotidase, is a glycosylated protein whose cDNA is homologous to that of adenosine triphosphate and diphosphate and can be secreted outside the cell. It is widely expressed in human tissues [4]. Studies have shown that CANT1 mutation is present in bone dysplasia (DD) and multiple epiphyseal dysplasia (MED), which are autosomal negative genetic diseases characterized by short metacarpal bones and long phalanges. The synthesis of proteoglycan in fibroblasts of patients with CANT1 mutation has been found, indicating that CANT1 plays a role in proteoglycan metabolism [5–7]. It was also found to be involved in tumor growth. CANT1 was overexpressed in prostate cancer, and associated with T status [8]. CANT1 was elevated in lung squamous cell carcinoma(LUSC), and knockdown of CANT1 blocked LUSC cell proliferation [9]. CANT1 expression was significantly increased in lung adenocarcinoma(LUAD), and was significantly associated with the T stage. Regression analysis considered CANT1 as a prognostic marker in LUAD [10]. CANT1 silencing suppressed clear cell renal cell carcinoma cell proliferation, migration, and invasion, which arrested the cell cycle in the S phase, and promoted apoptosis [11]. However, there is little research on the correlation between CANT1 and HCC. Therefore, our study aimed to investigate the expression of CANT1 in HCC and its potential clinical value.

The differential expression of CANT1 in HCC and normal tissues was analyzed using different data sets (TCGA and ICGC). The correlation between CANT1 expression and clinicopathology was studied, and its prognostic value was discussed, and its biological process was revealed. Finally, the relationship between CANT1 and immune cell infiltration was analyzed, and the mechanism of CANT1 promoting liver cancer development was comprehensively studied.

## Materials and methods

### Data preparation

Primary transcriptome sequencing data and clinical information of HCC patients were acquired from the Cancer Genome Atlas (TCGA) database, including the liver tissues of 374 patients and 50 normal people, as a training cohort. The expression level of the CANT1 gene was extracted by R software, and the difference between the two groups was analyzed based on the Wilcoxon test. In addition, the data on 33 kinds of cancers from UCSC Xena (https://xenabrowser.net/datapages/) was downloaded to analyze the CANT1 expression level for pan-cancer research. In addition, HCC samples were also obtained from ICGC (LIRI-JP), including 232 HCC patients, as a validation cohort. Moreover, we performed immunohistochemistry(IHC) on liver cancers from 53 HCC samples and 20 normal liver tissues collected from Beijing Ditan Hospital to confirm the CANT1 protein expression. Since no stage IV specimens were obtained, we only collected pathological stage I, stage II, and stage III. The patients were informed research content and signed an informed consent before the operation. The study was approved by the Institutional Research Ethics Committee of Beijing Ditan Hospital (batch number: NO.DTEC-KT2022-003-01).

### Immunohistochemistry(IHC)

The following steps were followed when performing IHC. After sections were dewaxed and hydrated, high-pressure antigen repairing was performed on them. Then, we blocked samples with goat serum and incubated them overnight with anti-CANT1 (Invitrogen, MA5-26752, Dilution 1:150). On the next day, a secondary antibody was added, and color development was carried out by a DAB chromogen kit. The IHC staining results were analyzed and scored by two pathologists who were blinded to the sources of the clinical samples. The intensity of staining was analyzed by the semiquantitative integration method, namely expression intensity multiplied by expression area. Expression intensity was scored from 0 to 3, and expression area was scored from 0 to 4. The results for CANT1 expression were then categorized based on score ranges of 1–3, 4–6, and 7–12, which were represented by +, ++ and +++, respectively.

### Correlation analysis of CANT1 expression with clinicopathological data

Based on the TCGA database and our Ditan cohort, we studied the correlation between CANT1 expression and clinicopathological data, including age, gender, T stage, N stage, M stage, and pathological stage using the Chisq test.

### Correlation analysis between CANT1 expression and prognosis

We analyzed the prognostic impact of CANT1 expression on HCC from TCGA and ICGC databases. Kaplan–Meier survival analysis, univariate and multivariate Cox analyses, and receiver operating characteristic (ROC) curve analysis were conducted using the “survival”, “survminer”, and “survivalROC” R packages.

### Analysis of CANT1 and DNA methylation

MethSurv (https://biit.cs.ut.ee/methsurv) is a web tool for survival analysis based on CpG methylation patterns, which utilizes 7358 methyl groups from 25 different human cancers from “TCGA” and the Cox proportional hazards model was used to develop an interactive web tool for survival analysis [12]. We assessed the OS of patients with CpG methylation in CANT1 based on the MethSurv database.

### Relationship between CANT1 and immune features

The TIMER database is an open public website which the immune cell infiltration in HCC with CANT1 expression was analyzed, including B cells, CD4 + T cells, CD8 + T cells, Neutrophils, Macrophages, and Dendritic cells. The infiltration level of 24 tumor immune cells in HCC samples was detected by the GSVA software package in the R and ssGSEA method. The relative enrichment of 24 immune cell types was scored, including B cells, T cells, macrophages, and neutrophils [13]. The correlation between CANT1 expression and immune cell infiltration level was evaluated by Spearman correlation analysis. The impact of immune cell infiltration and individual gene expression level on clinical outcomes was also analyzed based on the TIMER database [14]. The “survival” module was used to explore the clinical relevance of one or more tumor immune subsets and flexibly adjust for multiple covariates in a multivariable Cox proportional hazards model. Finally, the Wilcoxon test was performed to explore the correlation of CANT1 expression with the immune checkpoint gene levels.

### Drug sensitivity analysis

To explore the potential applicability of the CANT1 in clinical treatment decisions, the “pRRophetic” R package was used to evaluate the common chemotherapy or targeted drugs sensitivity [15]. Half-inhibitory concentration(IC50) is an important indicator for evaluating the efficacy of a drug or the response of a sample to treatment.

### Screening of differentially expressed genes (DEGs)

According to the TCGA database, we divided the expression of CANT1 in HCC patients into low-expression and high-expression group, and the differential expression genes(DEGs) was used to screen by the Deseq2 software package in R(Version 4.2.1). The threshold was | logFC |>1 and adjusted *P*<0.05 [16]. The volcano map of DEGs was constructed using the ggplot2 software package of R.

### Functional enrichment analysis and interaction analysis

To determine the biological function of CANT1 and its related DEGs, we annotated them with Gene Ontology (GO) and the Kyoto Encyclopedia of Genes and Genomes (KEGG) [17]. The GO analysis included the biology process (BP), molecular function (MF) and cell component (CC). Functional enrichment analysis was performed using clusterProfiler in R (version 4.2.1) [18]. Gene Set Enrichment Analysis (GSEA) is a computational method that determines whether a priori-defined set of genes shows statistical significance and concordant differences between two biological states [19]. The gene expression data were divided into groups with high and low CANT1 expressions according to the expression level of CANT1. Significant enrichment was defined as a normal *P*-value < 0.05 and FDR<0.25. String(https://string-db.org/) is a tool for studying the interaction network between proteins and helps to discover the core regulatory genes [20], which is used to construct the complex gene-gene functional interaction network of CANT1.

### Statistics Analysis

Wilcoxon test was applied to investigate CANT1 expression and its correlation with clinical characteristics according to the TCGA and GTEx database. Survival curves and logistic regression analysis were drawn using the R packages “survival” and “survminer”. The “clusterProfiler” in the R software (Version4.2.1 )was used to analyze GO, KEGG pathway, and GSEA enrichment. p < 0.05 indicates statistical significance.

## Results

### Pan-Cancer analysis of CANT1 expression and its association with clinical characteristics in HCC

**Fig. 1:**
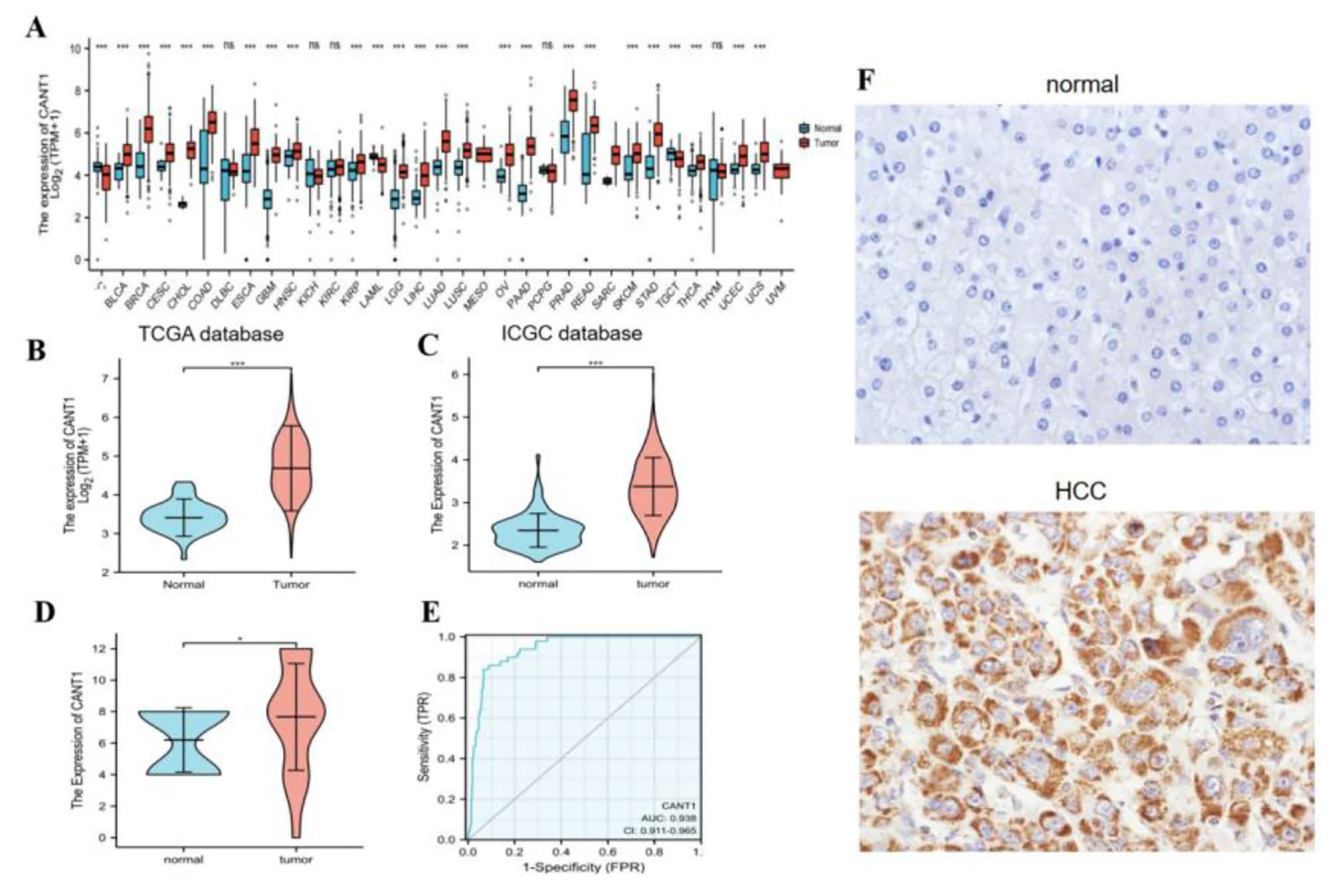
Expression of CANT1 in HCC and other different cancers. (**A**) The expression of CANT1 in pan-cancer tissues in TCGA database. (**B**, **C**) The expression of CANT1 in HCC tissues and normal tissues by TCGA and ICGC database. (**D**) The expression of CANT1 was higher in HCC than normal tissues in our HCC samples. (**E**) The ROC curve of diagnosis to distinguish HCC tissues from normal tissues . (**F**) The higher protein of CANT1 in HCC than normal tissues using immunohistochemistry. (Envision, Original magnificationX400). (***P < 0.001, **P < 0.01, *P < 0.05)

The RNA-seq expression data were downloaded from TCGA and GTEx to analyze the expression of CANT1 in 33 types of cancer. The results suggested that it was highly expressed in 22 cancers, including BLCA, BRCA, CESC, CHOL, COAD, ESCA, GBM, HNSC, KIRP, LGG, LIHC, LUAD, LUSC, OV, PAAD, PRAD, READ, SKCM, STAD, THCA, UCEC, and UCS. However, it was low expressed in ACC, LAML, and TGCT(Fig. 1A, B). Subsequently, the high expression of CANT1 was confirmed in another data set, such as LIRI-JP of ICGC (Fig. 1C). The area under the curve(AUC)of CANT1 was 0.938, indicating that the predictive ability of CANT1 has high accuracy(Fig. 1E). In addition, the expression level of CANT1 was significantly correlated with the T stage, pathological stage, and histological grade(Table 1). Other clinical features, such as age, gender, N stage, and M stage, showed no correlation with CANT1 expression(Table 1). Furthermore, in our Ditan cohort, we selected 53 samples of HCC and 20 normal tissues for the IHC study. The results suggested that the expression of CANT1 was higher in HCC tissues than in normal tissues(Fig. 1D, F). The protein expression of CANT1 was correlated with histological grade. However, other clinical features, such as age, gender, T stage, and pathological stage, displayed no relationship with CANT1 expression(Table 1).

**Table 1:** Correlation between CANT1 expression and clinical features in TCGA cohort and Ditan cohort

	Total	CANT1		χ²	*P*
		Low expression	High expression		
**TCGA cohort**					
**Age**					
<=60 years	177	81	96	2.578	0.109
> 60 years	166	106	90		
**Gender**					
Female	121	56	65	0.99	0.32
Male	253	131	122		
**T stage**					
T1 + T2	278	147	131	4.806	**0.028**
T3 + T4	93	37	56		
**N stage**					
N0	254	120	134	0.826	0.364
N1	4	1	3		
**M stage**					
M0	268	127	141	1.249	0.264
M1	4	3	1		
**Pathologic stage**					
I + II	260	138	122	5.421	**0.02**
III + IV	90	35	55		
**Histologic grade**					
G1 + G2	233	136	97	18.29	**< 0.001**
G3 + G4	136	48	88		
**Ditan cohort**					
**Age**					
<=60 years	36	7	29	1.57	0.3
> 60 years	17	6	11		
**Gender**					
Male	43	10	33	0.69	0.2
Female	10	3	7		
**Histologic grade**					
G1	12	7	5	9.58	**0.004**
G2 + G3	41	6	35		
**T stage**					
T1 + T2	45	10	35	0.86	0.39
T3 + T4	8	3	5		
**Pathologic stage**					
I	22	7	15	1.08	0.3
II + III	31	6	25		

### Evaluation of the Prognostic Value of CANT1 in HCC

**Fig. 2:**
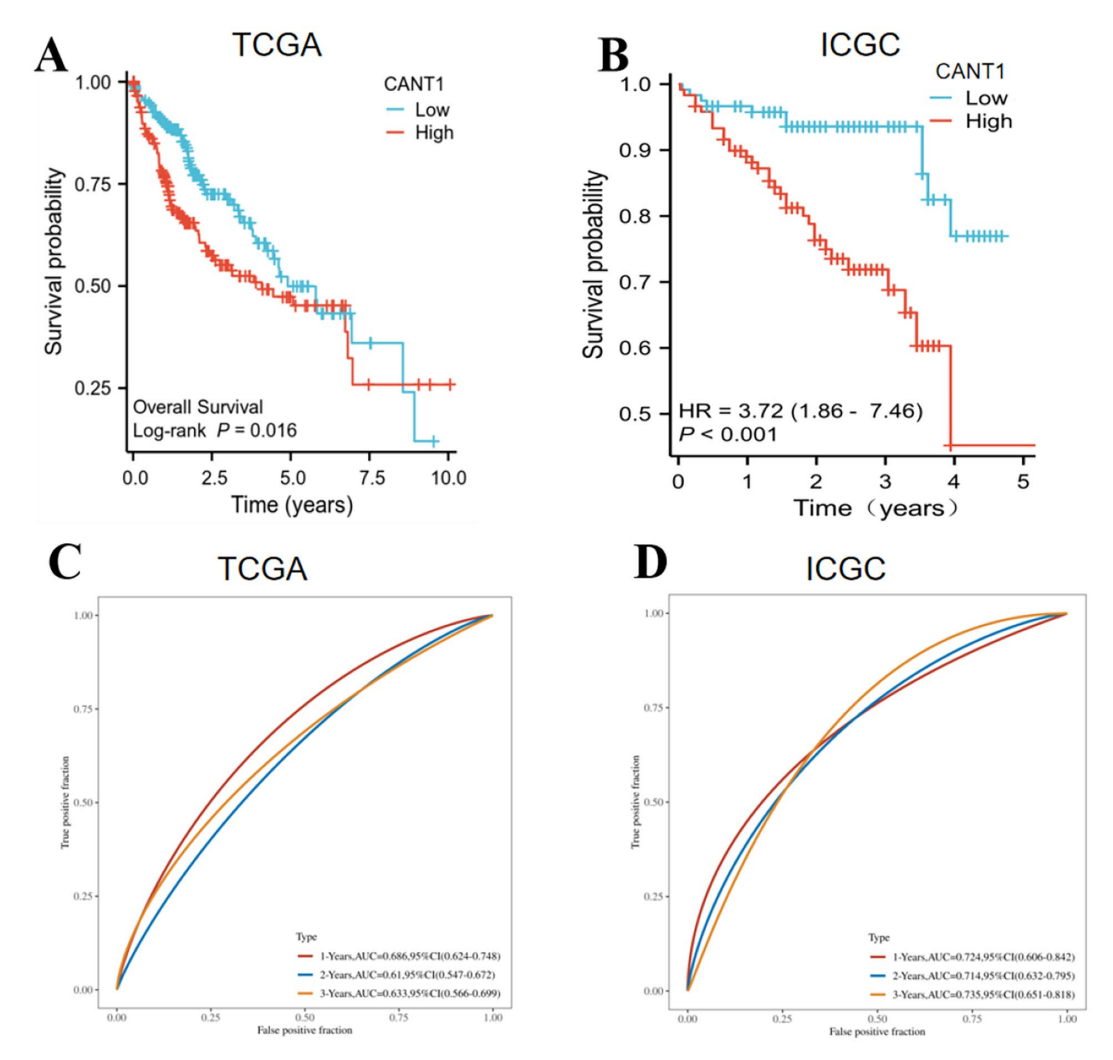
Prognostic value of CANT1 in HCC from different database; (**A**-**B**) Higher expression of CANT1 had poor OS in TCGA and ICGC database. (**C**-**D**) Time-dependent survival ROC curve analysis to predict 1-, 2- and 3-year survival rates in TCGA and ICGC database.

**Fig. 3:**
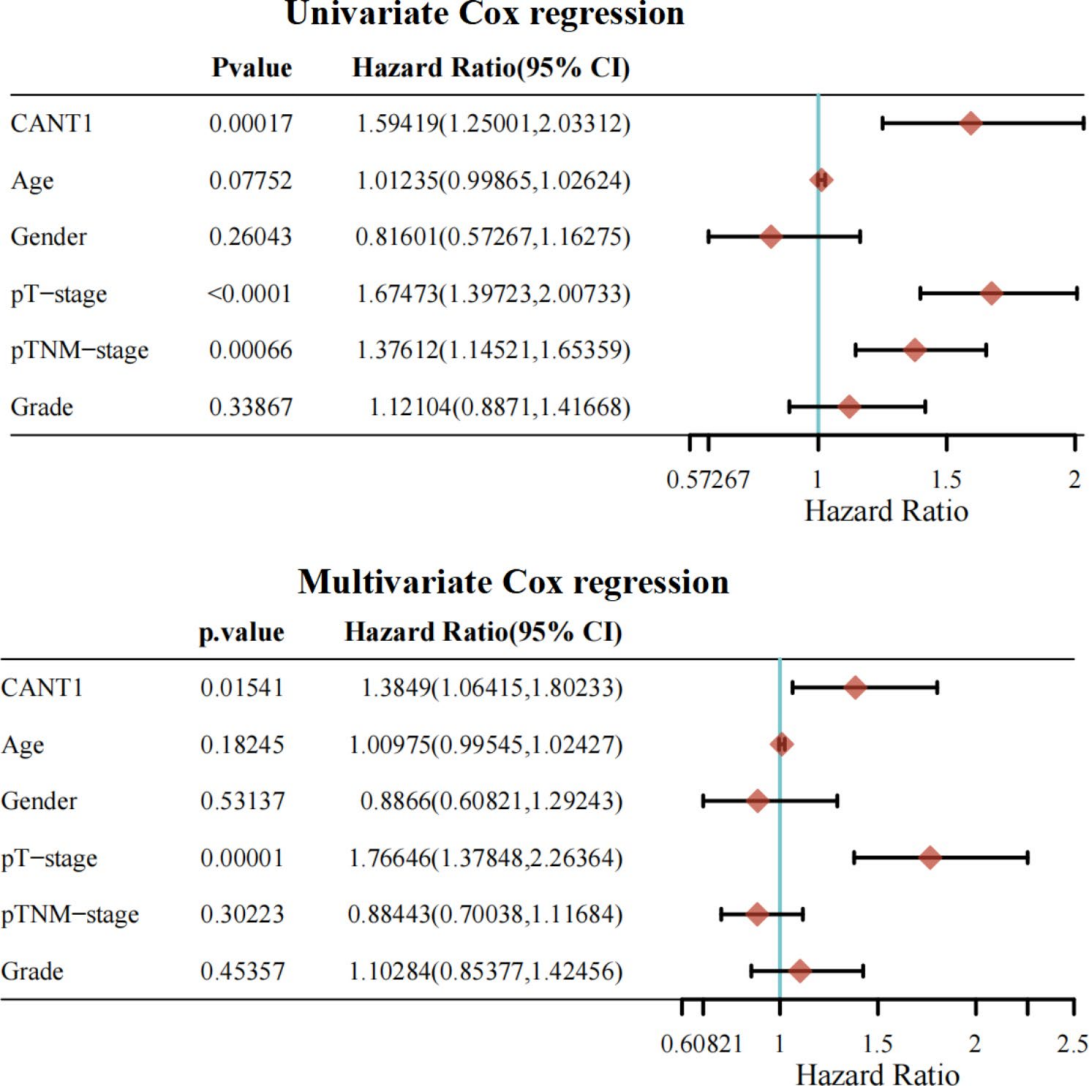
Univariate and multivariate Cox regression analyses of selected variables on OS in TCGA database.

The clinical data and CANT1 expression data of HCC patients were obtained from TCGA and ICGC databases. We investigated the relationship between CANT1 expression and OS of HCC patients. The patients were divided into low and high-expression groups based on the median value. According to the Kaplan-Meier plot, patients with a higher CANT1 expression showed poorer OS than those with a low expression in both training and validation cohorts (Fig. 2A, B). To evaluate the effectiveness of CANT1 as a biomarker for HCC, our team examined the AUC, with the AUC values at 1-, 2-, and 3-year survival being 0.686, 0.61, and 0.633, respectively(Fig. 2C). The prognostic value of CANT1 expression for HCC was further confirmed in the validation set(Fig. 2D). We adopted univariate and multivariate Cox regression to analyze the OS of patients with HCC and to study the independent predictive power. T stage, TNM stage, and the expression level of CANT1 were associated with OS of HCC patients according to the univariate analysis. In multivariate analysis, the T stage and expression level of CANT1 were related to OS of HCC patients (Fig. 3). In conclusion, CANT1 exhibited relatively good predictive accuracy in HCC patients.

### Prognostic value of CANT1 methylation in HCC patients

**Fig. 4:**
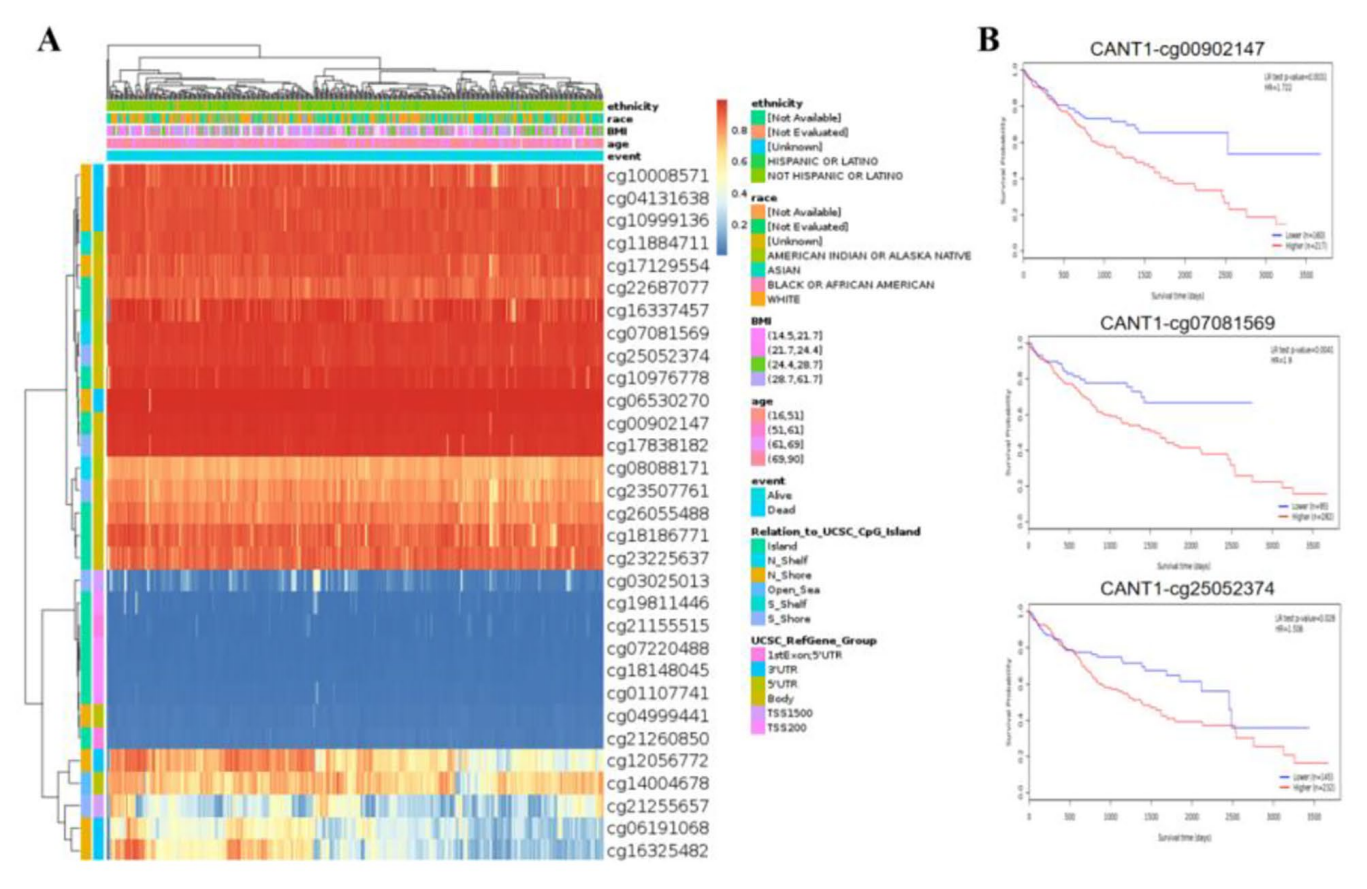
Heatmap of CANT1 expression and methylation levels (**A**), and the association between methylation sites in CANT1 with overall survival (**B**).

The MethSurv database results demonstrated that there were 31 CpG methylation sites on CANT1. As shown in Figs. 4A, 13 highly methylated sites can be seen, including cg10008571, cg04131638, cg10999136, cg11884711, cg17129554, cg22687077, cg16337457, cg07081569, cg25052374, cg10976778, cg06530270, cg00902147, and cg17838182. Among them, three methylation sites(cg00902147, cg07081569, and cg25052374) were associated with prognosis. The patients with CANT1 hypomethylation at cg00902147, cg07081569, and cg25052374, had better OS(Fig. 4B).

### Correlation analysis between CANT1 and immune features

**Fig. 5:**
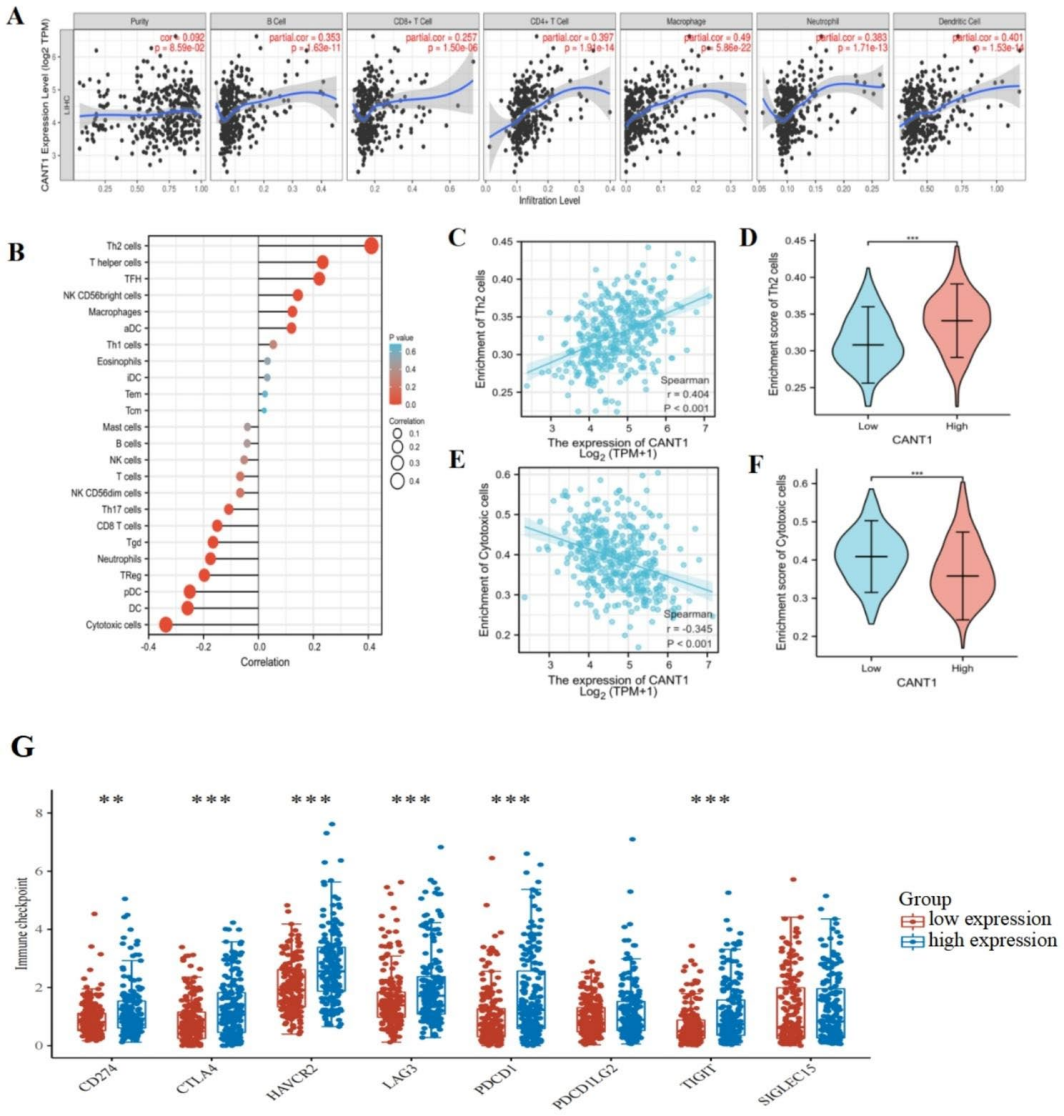
Relationship between CANT1 expression and immune cell infiltration. (**A**) Relationship between CANT1 expression and immune cell infiltration in TIMER database. (**B**) The lollipop chart of immune cells infiltration and CANT1 by ssGSEA. (**C**-**D**) The positive correlation between CANT1 expression and enrichment of Th2 cells. (**E**-**F**) The negative correlation between CANT1 expression and enrichment of cytotoxic cells. (**G**) The correlation between CANT1 expression and immune checkpoints. (***P < 0.001, **P < 0.01, *P < 0.05)

The relationship between CANT1 and immune cell infiltration was studied based on TIMER. The results showed that CANT1 expression was positively correlated with B cells, CD4 + T cells, CD8 + T cells, neutrophils, macrophages, and dendritic cells, but not with purity(Fig. 5A). Subsequently, the ssGSEA algorithm and Spearman correlation were used to show the correlation between the expression level of CANT1 and the level of immune cell infiltration in the HCC tumor microenvironment. The results demonstrated that CANT1 expression was positively correlated with infiltration levels of Th2 cells, T helper cells, TFH, NK CD56 bright cells, Macrophages and aDC, and was negatively correlated with CD8 T cells, Tgd, Neutrophils, Treg, cytotoxic cells, pDC, and DC(Fig. 5B). The level of Th2 cell infiltration was significantly positively correlated with CANT1 expression (Fig. 5C-D). In contrast, the infiltration level of cytotoxic cells was significantly negatively correlated with CANT1 expression (Fig. 5E-F), and was lower in the CANT1 high expression group compared with the low expression group. These results suggested that CANT1 might be involved in the immune infiltration of HCC. In addition, the independent prognostic value of immune cell infiltration and CANT1 expression were analyzed using Cox proportional risk regression. The results showed that the expression of CANT1 and the infiltration degree of the four immune cells(B cell, CD8 T cell, Macrophage, and Dendritic) were independently associated with significantly shortened OS(Table 2). In recent years, more and more studies have confirmed that immune checkpoint inhibitors can eliminate the immune evasion effect of liver cancer cells by releasing the immune suppression function of immune checkpoints.The application of immune checkpoint inhibitors has become the latest means of antitumor therapy. We investigated the correlation between CANT1 expression and immune checkpoints. The expression of immune checkpoints, such as CD274 (PD-L1), CTLA4, HAVCR2, LAG3, PDCD1 (PD-1) and TIGIT, was higher in the CANT1 high-expression group than that in the CANT1 low-expression group(Fig. 5G). The above results suggested that CANT1 may play an important role in tumor immunity.

**Table 2:** The Cox proportional hazards regression of immune cell infiltration and CANT1 expressions.

	coef	HR	95%CI_l	95%CI_u	p.value	sig
Age	0.014	1.014	0.998	1.03	0.088	
Race Black	0.67	1.954	0.776	4.92	0.155	
Race White	0.068	1.07	0.701	1.633	0.754	
B_cell	-9.023	0	0	0.169	0.015	*
CD8_T cell	-5.182	0.006	0	0.802	0.041	*
CD4_T cell	-5.457	0.004	0	2.446	0.092	
Macrophage	5.116	166.621	1.029	26976.181	0.049	*
Neutrophil	-2.067	0.127	0	12704.224	0.725	
Dendritic	4.758	116.543	3.252	4176.945	0.009	*
CANT1	0.457	1.58	1.156	2.159	0.004	*

### Drug sensitivity analysis

**Fig. 6:**
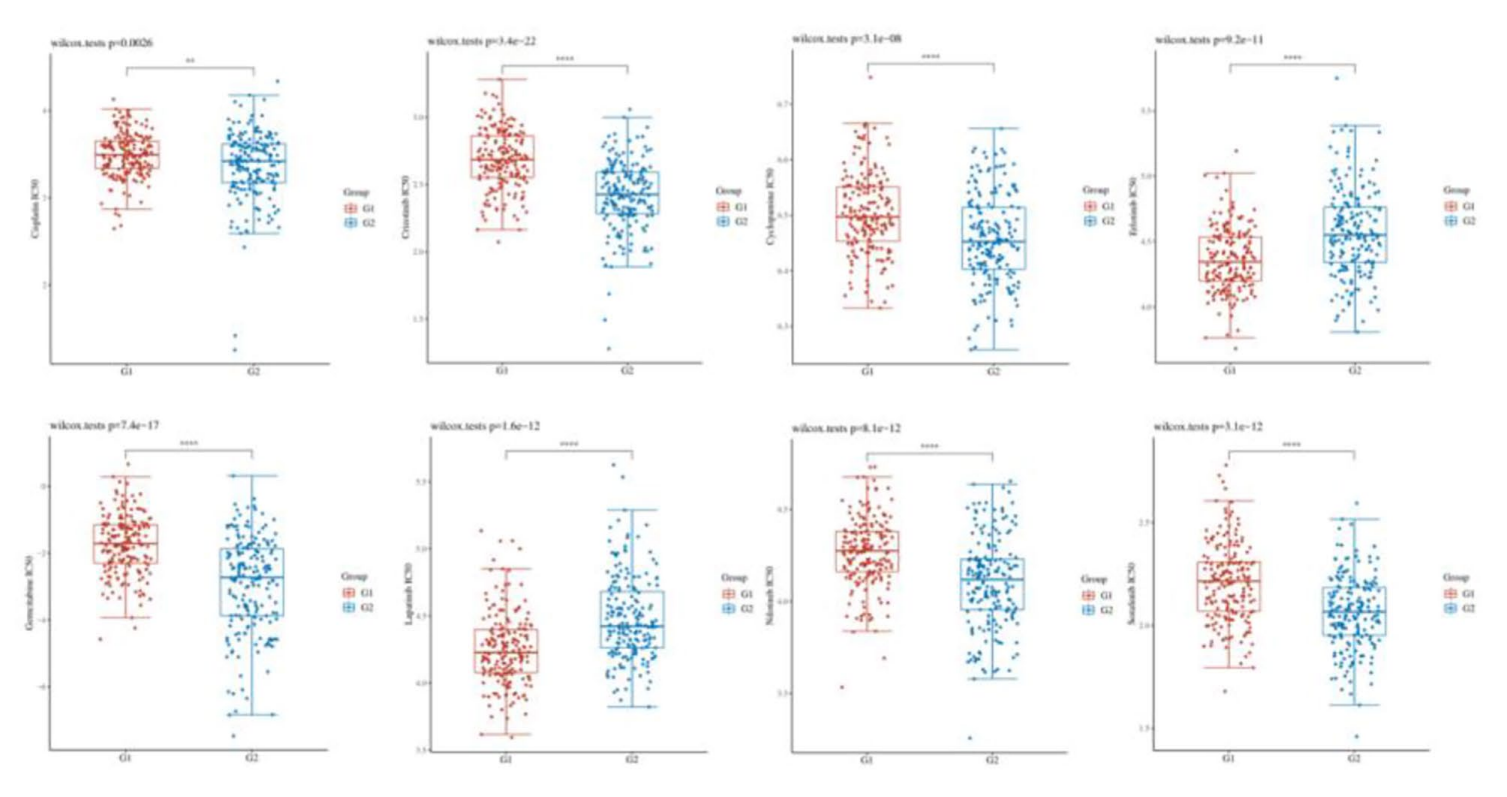
The correlation between CANT1 expression and drug sensitivity. (G1: low expression; G2: high expression; ***P < 0.001, **P <  0.01,*P < 0.05)

We evaluated chemotherapeutic drugs for HCC using IC50. Patients with lower IC50 values are more sensitive to the drug. We found that the IC50 value of Cisplatin, Crizontinib, Cyclopamine, Erlotinib, Gemcitabine, Lapatinib, Nilotinib, Sorafenib in CANT1 high-expression group had lower than in low-expression group, indicating that the patients with CANT1 overexpression were more sensitive to these drugs (Fig. 6).

### Identification and Functional Analysis of DEGs

**Fig. 7:**
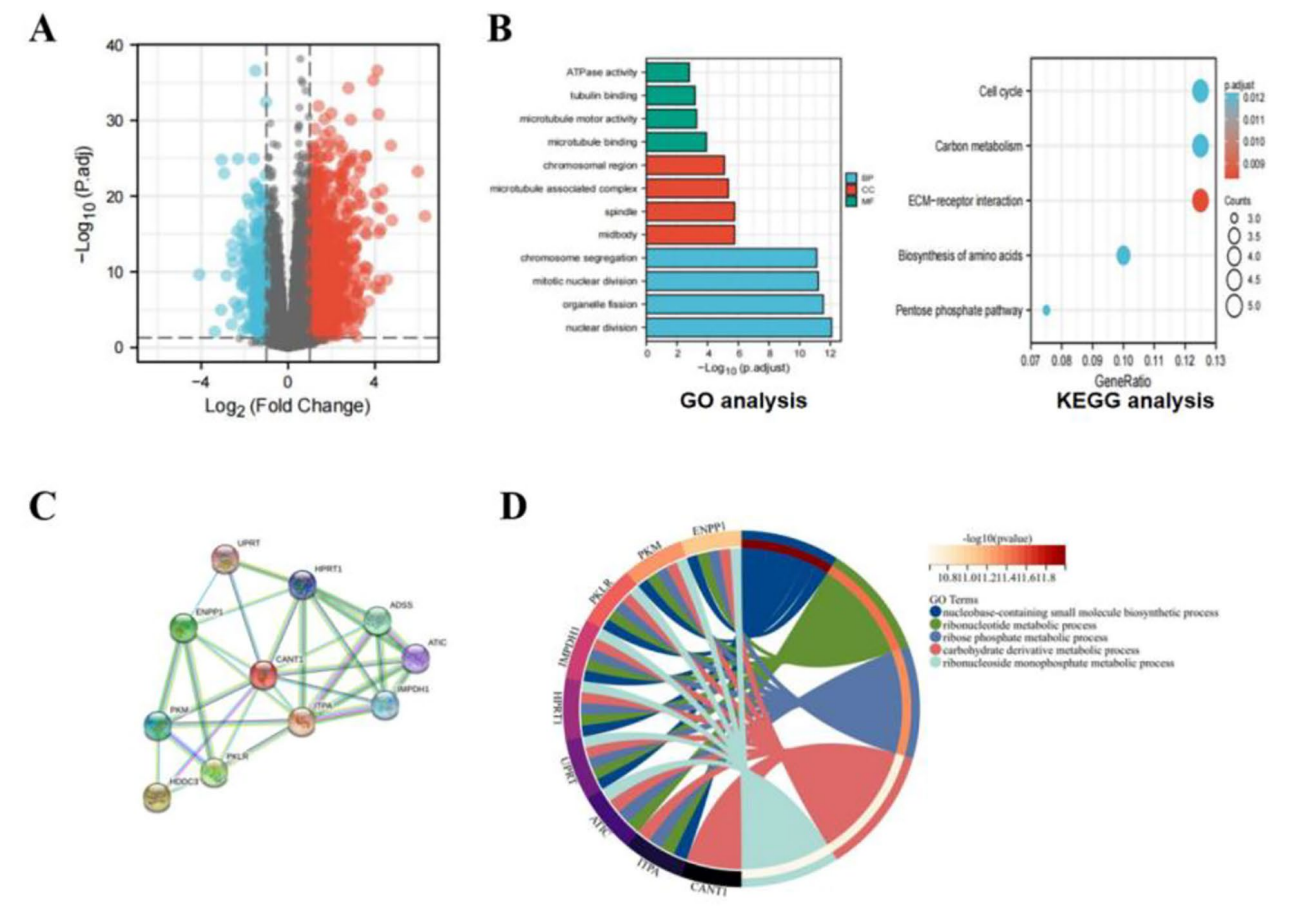
Identification and functional enrichment analysis of the differentially expressed genes (DEGs) between high and low CANT1 expression groups. (**A**) The volcano plot of DEGs. (**B**) The GO and KEGG enrichment of 100 up-regulated DEGs. (**C**) The PPI of CANT1from String database. (**D**) GO term enrichment analysis.

A total of 1398 up-regulated genes and 343 down-regulated genes were obtained based on log2FC>1, *P*. adj<0.05. The volcano map of DEGs in HCC and normal tissues is shown in Fig. 7A. The GO and KEGG analyses were conducted on the first 100 up-regulated genes, and the results revealed that the BP of DEGs was mainly enriched in the nuclear division, organelle fission, mitotic nuclear division, and chromosome segregation. For MF, these genes mainly focused on microtubule binding, microtubule motor activity, tubulin binding, and ATPase activity. The CC of DEGs enrichment was concentrated in the midbody, spindle, microtubule associated complex, and chromosomal region. The results of KEGG enrichment suggested that DEGs participated in the cell cycle, carbon metabolism, ECM receptor interaction, biosynthesis of amino acids, and pentose phosphate pathway (Fig. 7B). Moreover, according to the PPI network, CANT1 interacted with UPRT, HPRT1, ADSS, ATIC, IMPDH1, ITPA, PKLR, HDDC3, PKM, and ENPP1, which mainly participated in nucleobase-containing small molecule biosynthesis, ribonucleotide metabolism, ribose phosphate metabolism, etc. (Fig. 7C, D).

**Fig. 8:**
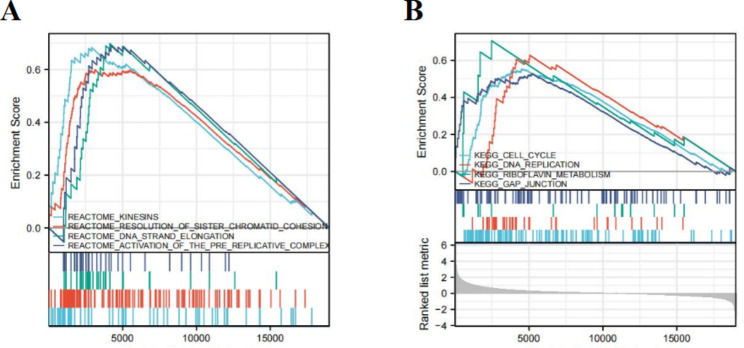
Gene set enrichment analysis(GSEA) of high CANT1 expression phenotype in HCC based on Reactome (**A**) and KEGG pathways (**B**)

To further reveal the potential pathway that CANT1 might regulate the carcinogenesis and development of HCC, GSEA analysis was carried out on CANT1-related DEGs and demonstrated the positive correlation of CANT1 with kinesin, resolution of sister chromatid cohesion, DNA strand elongation, and activation of the pre-replication complex based on the Reactome pathway(Fig. 8A). CANT1 overexpression was enriched in the cell cycle, DNA replication, riboflavin metabolism, and gap junction based on the KEGG pathways (Fig. 8B). These results indicated that CANT1 exerted an influence on the occurrence and development of tumors through the above enrichment pathways.

## Discussion

Because of its complicated molecular mechanism, liver cancer has been one of the most dangerous malignant tumors globally. Liver cancer is characterized by rapid growth, vascular invasion, and high tolerance to chemotherapy and targeted therapies [21, 22]. Although the upgraded liver cancer detection and treatment increases the possibility of cure, the prognosis of liver cancer is still poor due to postoperative recurrence or metastasis [23]. In recent years, the development and maturity of whole genome sequencing technology have provided great help for the exploration of new biomarkers and new therapeutic targets. However, further exploration is required in the early diagnosis of HCC and prognostic molecular targets.

CANT1 is considered to be a glycosylated protein, and its encoded protein belongs to the phosphatase family. It was overexpressed in lung cancer [24], prostate cancer [8], and clear cell renal carcinoma (ccRCC) [11]. In lung cancer, CANT1 promoted the progression of lung cancer by driving the nuclear factor-k gene binding (NF-κB) signaling pathway. Moreover, a significant difference existed between the expression level of CANT1 and N stage, and the highly expressed CANT1 had a poor prognosis. In squamous cell lung carcinoma [9], mir-607/CANT1 played an important role in its progression by mediating the EMT process, and was associated with poor prognosis. In clear cell renal carcinoma, CANT1 silencing inhibited the proliferation, migration, and invasion of cells. Chen Yang et al. screened out three candidate genes in HCC with TP53 mutation, including CANT1. Many studies have demonstrated that HCC patients with mutated TP53 have adverse prognosis, which provided new insights into personalized prognostication approaches [25].The present study analyzed CANT1 expression in 33 tumors by downloading RNAseq from TCGA and GTEx databases, and revealed high expression of CANT1 in some tumors, including HCC. The high expression of CANT1 in HCC tissues was confirmed in different databases (i.e. ICGC). IHC staining showed that CANT1 expression was higher in HCC samples than in normal tissues.

We further studied the relationship between CANT1 expression level and clinicopathological features of HCC patients. CANT1 expression was significantly correlated with T stage, pathologic stage, and histologic grade in the TCGA database. Our Ditan cohort showed that CANT1 expression was only correlated with histologic grade, not pathological stage and T stage, probably due to the small number of cases we collected and the inability to collect stage IV cases. Kaplan-Meier curves indicated that patients with high CANT1 expression presented poor OS in training and validation cohorts. Univariate and multivariate Cox regression analysis also identified that CANT1 expression level was an independent prognostic biomarker of OS in HCC patients. Furthermore, we predicted time-dependent survival ROC curve of 1-, 2-, and 3-year, with AUCs greater than 0.6 in TCGA and ICGC sets. These results suggested CANT1 as a potential prognostic marker for HCC.

Increasing evidence shows that epigenetic changes, especially abnormal DNA methylation, could lead to transcriptional silencing of tumor suppressor genes or high expression of oncogenes, thus deteriorating malignant tumors [26]. Aberrant DNA methylation resulted in malignant tumors mainly by DNA hyper- or hypo-methylation [27]. We investigated the relationship between the methylation level of CANT1 and prognosis, and three CpG sites were associated with prognosis, and the three CpG sites with hypomethylation had better OS. Immune cell infiltration and tumor microenvironment(TME) have been proven to play a key role in cancer progression [28]. Tumor immunity has been a hot topic in recent years [29]. Immunotherapy may be a promising anti-liver cancer strategy. TME is the environment in which tumor cells grow and plays an important role in the occurrence and development of tumors, which is involved in the process of tumor malignant transformation, tumor growth, metastasis and drug resistance [30]. Immune cell infiltration accelerated the progression of chronic hepatitis to liver cancer, and was associated with a poor prognosis of liver cancer [19, 31, 32]. In our study, CANT1 was positively associated with the infiltrations of six immune cells (B cells, CD4 + T cells, CD8 + T cells, neutrophils, macrophages and dendritic cells). In addition, ssGSEA was used to study the relationship between CANT1 and 24 types of immune cell infiltration. The results demonstrated a significant weak-to-moderate positive correlation of CANT1 expression with Th2, T helper cell, TFH, NK CD56 bright cells, Macrophages and aDC. Moreover, the infiltration level of Th2 was highest in high expression CANT1. This was possibly attributed to Th1/Th2 imbalance induced by high expression of CANT1 as well as the inhibitory effects of Th2-generated cytokines on Th1 proliferation and differentiation and the function of cytotoxic T lymphocytes, resulting in immunosuppression and the occurrence and development of tumors [33]. Furthermore, CANT1 expression was negatively correlated with Cytotoxic cell, DC, pDC, Treg, Neutrophils,Tgd and CD8 T cells. Cytotoxic cells have the function of killing cancer cells and have anti-tumor activity. CANT1 overexpression leads to downregulation of cytotoxic cells, which leads to the occurrence of liver cancer. Dendritic cells(DC) are the strongest specialized antigen presenting cells and play a unique role in anti-tumor immunity. Overexpression of CANT1 leads to downregulation of DC, thus limiting the activity of effector T cells and promoting tumor growth. All these suggested that CANT1 might alter the immune microenvironment of HCC and impact immune regulation. In addition, Cox proportional risk models showed that B cells, CD8 + T cells, macrophages, and dendritic cells were significantly associated with adverse clinical outcomes in HCC patients. In recent years, the discovery of immune checkpoints such as PD-1, PD-L1 and CTLA4 and the development and application of corresponding immune checkpoint inhibitors have provided new options for the treatment of advanced liver cancer [34]. In HCC, high PD-L1 expression is usually associated with high PD-1 expression, and associated with cancer recurrence, metastasis, and a high risk of cancer-related death [35, 36]. CTLA4 is one of the T cell receptors and can competitively prevent CD28 from binding to B7, thereby inhibiting T cell activation [37, 38]. Our study found that the expression of CANT1 is associated with the expression of most immune checkpoints, such as PD-1, PD-L1 and CTLA4. These findings suggested that CANT1 may be involved in immunity, which may provide new insights into the immunotherapy of HCC. Finally, we studied the relationship between CANT1 expression and drug sensitivity, and the results showed that eight drugs with notable expression differences were extracted, namely Sorafenib, Cisplatin, Crizontinib, Cyclopamine, Erlotinib, Gemcitabine, Lapatinib, and Nilotinib, which provided the theoretical basis for clinical medication.

To further understand the biological function of CANT1 in HCC, we identified DEGs between HCC patients with high- and low- expression of CANT1. GO and KEGG enrichment analysis was performed for 100 up-regulated genes, showing that DEGs were mainly enriched in cell cycle, nuclear division and microtubule binding. In addition, GSEA analysis demonstrated that CANT1 chiefly participated in the cell cycle, DNA replication, riboflavin metabolism, and gap junction. These enriched pathways were related to the mechanisms of the tumor.

We first explored the relationship between CANT1 and HCC, certain limitations were unavoidable: all data analyzed by bioinformatic methods were directly downloaded from public databases, and the protein expression level of CANT1 was only verified by IHC. Research is also needed at RNA levels and oncogenic mechanism in HCC through in vitro and in vivo experiments.

## Conclusions

In conclusion, the CANT1 expression level was elevated in HCC and had close ties to a poor prognosis. In addition, CANT1 could be involved in the occurrence and development of HCC by influencing the infiltration level of immune cells. On this basis, our study identified CANT1 as a latent prognostic biomarker in HCC. At present, the clinical significance and mechanism of CANT1 in HCC have not been reported.

## Data Availability

This research recruited public databases and website tools. The data is available here: TCGA(https://portal.gdc.cancer.gov), UCSC Xena (https://xenabrowser.net/datapages/), TIMER2 database (http://timer.comp-genomics.org/), and MethSurv (https://biit.cs.ut.ee/methsurv).

## Abbreviations

ACC: Adrenocortical carcinoma
BLCA: Bladder Urothelial Carcinoma
BRCA: Breast invasive carcinoma
CESC: Cervical squamous cell carcinoma and endocervical adenocarcinoma
CHOL: Cholangio carcinoma
COAD: Colon adenocarcinoma
DLBC: Lymphoid Neoplasm Diffuse Large B-cell Lymphoma
ESCA: Esophageal carcinoma
GBM: Glioblastoma multiforme
HNSC: Head and Neck squamous cell carcinoma
KICH: Kidney Chromophobe
KIRC: Kidney renal clear cell carcinoma
KIRP: Kidney renal papillary cell carcinoma
LAML: Acute Myeloid Leukemia
LGG: Brain Lower Grade Glioma
LIHC: Liver hepatocellular carcinoma
LUAD: Lung adenocarcinoma
LUSC: Lung squamous cell carcinoma
MESO: Mesothelioma
OV: Ovarian serous cystadenocarcinoma
PAAD: Pancreatic adenocarcinoma
PCPG: Pheochromocytoma and Paraganglioma
PRAD: Prostate adenocarcinoma
READ: Rectum adenocarcinoma
SARC: Sarcoma
SKCM: Skin Cutaneous Melanoma
STAD: Stomach adenocarcinoma
TGCT: Testicular Germ Cell Tumors
THCA: Thyroid carcinoma
THYM: Thymoma
UCEC: Uterine Corpus Endometrial Carcinoma
UCS: Uterine Carcinosarcoma
UVM: Uveal Melanoma
